# Effect of *Bursaphelenchus xylophilus* infection on leaf photosynthetic characteristics and resource‐use efficiency of *Pinus massoniana*

**DOI:** 10.1002/ece3.2642

**Published:** 2017-04-06

**Authors:** Ruihe Gao, Zhuang Wang, Juan Shi, Youqing Luo

**Affiliations:** ^1^ Beijing Key Laboratory for Forest Pest ControlBeijing Forestry UniversityBeijingChina

**Keywords:** *Bursaphelenchus xylophilus*, carbon isotope ratios, gas exchange, photosynthesis, *Pinus massoniana*

## Abstract

Pine wilt disease (PWD) is considered as the most destructive forest‐invasive alien species in China. We measured gas exchange parameters and foliar carbon isotope ratios (δ^13^C) of different infection phases of Masson pine in order to investigate the effect of *Bursaphelenchus xylophilus* infection on photosynthetic responses and resource‐use efficiency. The results showed that net photosynthetic rate (*P*_n_), transpiration rate (*T*), stomatal conductance (*g*_s_), and internal CO_2_ concentrations (*C*_i_) decreased in the infested trees at photosynthetic photon flux density (PPFD) levels from 0 to 2,000 μmol m^−2^ s^−1^ compared with controls. The maximum net photosynthetic rate (*P*_max_) was significantly declined in the infected trees than in controls (*p *<* *.05). There also exist significant differences in dark respiration rate (*R*_d_) among different infection phases (*p *<* *.05), but the value is highest in the middle infection phase, followed by the control and then the terminal infection phase. This indicates that *Pinus massoniana* plants need to consume more photosynthetic products during the middle infection phase in order to defend against pine sawyer beetle feeding and PWD infection. Isotopic analysis revealed a significant decrease of the foliar δ^13^C (*p *<* *.05), as much as 2.5‰ lower in the infected trees. The mean leaf N content was about 12.94% less in the middle infection phase and 27.06% less in the terminal infection phase, causing a significant increase of the foliar C:N ratio in infested trees. Both of the net photosynthetic rates and foliar δ^13^C were linearly correlated with the foliar N content. We also found a significant decrease (*p *<* *.05) of resource‐use efficiency in PWD‐induced *P. massoniana* plants, which can be attributed to the closure of stomatal pores and the inactivation or loss of both Rubisco and other key Calvin cycle enzymes. This study highlights the impact of photosynthetic characteristics, foliar carbon isotope ratios, and resource‐use efficiency of PWD‐induced trees, which can help identify PWD infestations at the photosynthetic and physiological levels so as to better facilitate management actions.

## 1 Introduction

**Figure 1:**
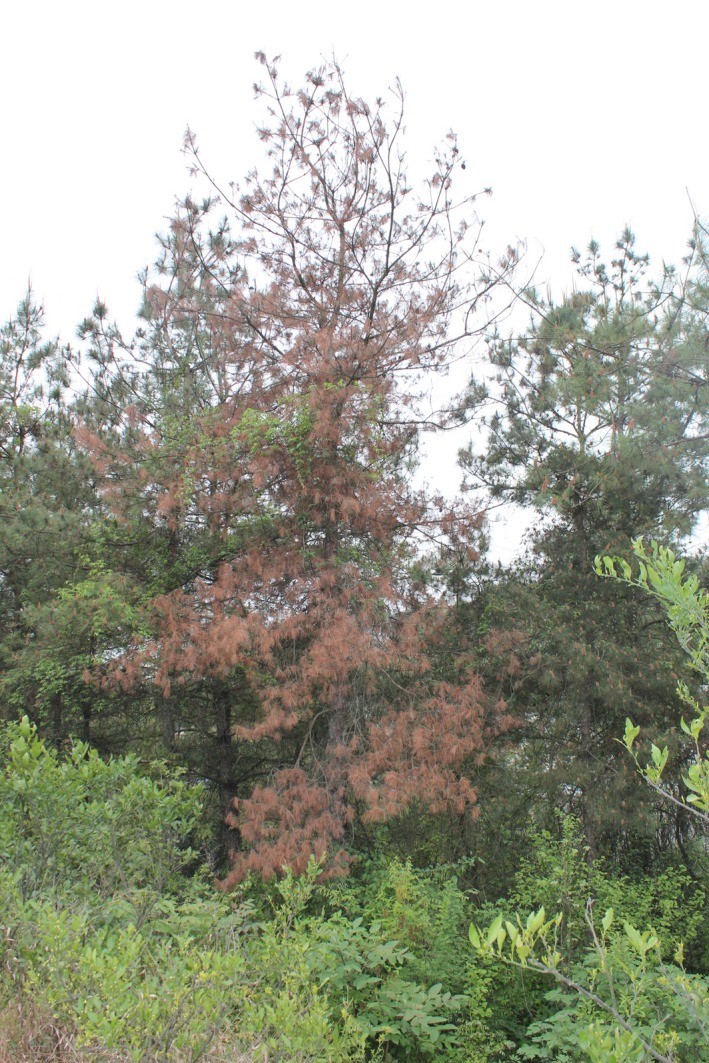
The infected Masson pine tree (*Pinus massoniana* Lamb.)

Pine wilt disease, which is caused by the pine wood nematode *Bursaphelenchus xylophilus* (Steiner and Buhrer) Nickle (Nematoda: Aphelenchoididae), was first introduced to China in 1982 and identified as the leading forest pest and disease (Shi et al., 2006; Wan, Zheng, & Guo, 2005). What is worse, nearly all the native pine species are highly susceptible to the invasion of *B. xylophilus* within the current PWD distribution areas in China. Masson pine (*Pinus massoniana* Lamb), belonging to the Pinaceae family, is a native species in China and can grow well in dry, sandy soils and arid climates (Zhao, Futai, Sutherland, & Takeuchi, 2008). *Pinus massoniana* is considered as the pioneer tree species for afforestation and is widely distributed across 19 provinces in the central and southern parts of China (Gao, Shi, Huang, Wang, & Luo, 2015). Green needles of *P. massoniana* trees affected by PWD turn to red and wilt (Figure 1), leading to the rapid death of the host tree. In China, it is difficult to completely eradicate this disease because pines will die within 2–3 months after being infected by *B. xylophilus*. The problem is exacerbated by a lack of effective control measures for the spread of pine wood nematodes (Gao et al., 2015; Hu et al., 2012). Therefore, the *B. xylophilus* invasion has caused huge economic losses in the timber industry as well as important ecological consequence for the forest ecosystem (Gao et al., 2015; Shi et al., 2007; Yu, Xu, & Ding, 2011).

In China, the pine sawyer beetle (*Monochamus alternatus* Hope) is the vector of *B. xylophilus* and can facilitate the nematode to spread from infected host trees to the healthy ones. When it arrives on a healthy tree, *B. xylophilus* can enter the resin canals through the broken canals in the cortex and xylem caused by the feeding pine sawyer beetles. Then, they can rapidly spread inside the infected hosts, up to 150 cm per day (Zhao et al., 2008). In addition, *B. xylophilus* can also exude cellulases and pectinases in order to kill the contents of living cells in the host trees (Kikuchi, Jones, Aikawa, Kosaka, & Ogura, 2004). This rapid diffusion combined with the fast reproduction of *B. xylophilus* leads to the dysfunction of the xylem and tracheid, which blocks the sap ascent and water transportation from the roots to the crown (Zhao et al., 2008).

In plants, infection by pests or diseases leads not only to the induction of defense systems but also to changes in photosynthetic activity. The reaction of host photosynthesis is an important physiological parameter and very sensitive to external stimulation, such as the invasion of pests, disease, and climate changes. To better understand the host reaction to the development of pine wilt disease, measurements of the changes in physiology and photosynthetic characteristics of the host are important (Zhao et al., 2008). Previous studies have demonstrated that the host's resistance mechanism to the *B. xylophilus* is a change in photosynthesis (Fukuda, 1997; Melakeberhan, Toivonen, Vidaver, Webster, & Dube, 1991; Woo et al., 2010). Woo et al. quantified decreases in the instantaneous gas exchange rate in the seedlings of *Pinus densiflora* inoculated with *B. xylophilus*. However, quantification of the continuous photosynthetic responses and resource‐use efficiency with certain environmental variables, such as CO_2_ or photosynthetic photon flux density (PPFD), can certainly provide a clearer picture of PWD‐induced photosynthetic activity.

Foliar carbon isotope ratio (δ^13^C) is an important indicator of physiological responses and can reflect the overall trade‐offs between carbon gain and water loss for plants (Querejeta, Barea, Allen, & Antonio, 2003; Walia, Guy, & White, 2010). Previous studies have confirmed that the foliar δ^13^C was highly correlated with intrinsic water‐use efficiency in C_3_ plants (Farquhar, Ehleringer, & Hubick, 1989; Meinzer, Woodruff, & Shaw, 2004; Querejeta et al., 2003; Walia et al., 2010). Furthermore, it can also be used to evaluate the covariation between photosynthetic capacity, carbon gain, leaf nitrogen content, etc. (Dawson, Mambelli, Plamboeck, Templer, & Tu, 2002; Flower, Knight, Rebbeck, & Gonzalez‐Meler, 2013).

The plant resource‐use efficiencies, which are influenced by light, temperature, humidity, water stress, and environmental stimulation (Field, Merino, & Mooney, 1983), change dramatically during the various life stages of an individual plant, as well as between different plant species. In addition, Dawson et al. (2002) suggested that carbon, water, and nitrogen are three of the most important resources that have a significant positive correlation with plant function, growth, and the biogeochemical cycles in which plants participate. In the case of PWD, relatively little is known about the impact of the infection on resource‐use efficiency of the host trees.

Although many studies have been conducted on the infection symptom, wilting mechanism, vector, and the biology and physiology of *B. xylophilus* (Gao et al., 2015; Kim et al., 2010; Mamiya, 1988; Shi, Chen, Luo, Wang, & Xie, 2013; Yoshimura et al., 1999), relatively little is known about the underlying impact of photosynthetic characteristic and resource‐use efficiency on PWD‐induced tree mortality. The primary objective of this study was to further determine the effects of the invasion of *B. xylophilus* on the photosynthetic characteristic and resource‐use efficiency of *P. massoniana* trees. In this study, our hypothesis was that *P. massoniana* trees infected with PWD would undergo a decrease in photosynthesis and resource‐use efficiency decreases during the infection by PWD compared with uninfected trees. The photosynthetic response of *P. massoniana* with various PWD infection phases were measured to determine the changes in CO_2_ assimilation, transpiration behavior, stomata condition, stable carbon isotopic composition, and resource‐use efficiency.

## 2 Methods

This study was taking place in the Yiling District of China, an eastern part of the Three Gorges reservoir region. With an eastern mid‐subtropical monsoon climate, the Yiling District has a mean annual precipitation and mean annual temperature of 997–1,370 mm and 16.6°C, respectively. *P. massoniana* is the primary coniferous tree species in this district. Since its first occurrence in 2006, *B. xylophilus* has spread rapidly in Yiling District.

The research was conducted in a 3‐year PWD‐infected Masson pine forest plots, which the stand density was 1,304 tree/ha and the basal area is 14.96 m^2^/ha. All *P. massoniana* trees in our plots were surveyed for *B. xylophilus* infection using Zhao's PWD rating system (Zhao et al., 2008), which is based on the external symptoms and internal changes of the *B. xylophilus‐*infected pine trees; it includes the following phases: control, initial infection phase, early infection phase, middle infection phase, serious infection phase, and terminal infection phase. For this project, we measured the control (healthy Masson pine), middle infection (discoloration of old needles, decrease of oleoresinosis, partial necrosis of cells, low conductivity of sap ascent, and high population of pine wood nematode), and terminal infection phases (discoloration of both old and young needles, none of oleoresinosis, wide‐area necrosis of cells, completely stop of sap ascent, and extensive propagation of pine wood nematode) of PWD‐induced *P. massoniana* trees. A total of nine large trees (three for each treatment, Table 1) were selected for measurements of photosynthetic gas exchange characteristics and carbon isotope ratios.

**Table 1:** Selected Masson pine sample trees for the measurement of photosynthetic characteristics, nutrient content, and isotope abundance

Trees	DBH (cm)	Height (m)	Age (year)
Control	14.5	8.54	22
	15.1	8.80	20
	16.0	9.10	25
Middle phase	13.5	8.06	21
	17.1	8.90	26
	15.0	8.32	23
Terminal phase	17.6	10.80	32
	18.7	9.76	28
	15.4	10.10	24

### 2.1 Leaf gas exchange

The measurements were conducted during the sunny days in August in 2014. Two branches were selected for each sampled Masson pine tree, and three replicate photosynthetic measurements were conducted per branch. The *P*_n_, *T*,* g*_s_, and *C*_i_ were measured with a portable photosynthesis system equipped with a red/blue LED source and CO_2_ injector (LI‐6400 XT, Li‐Cor Inc, USA). A clamp‐on leaf cuvette that exposed 6 cm^2^ of leaf area was equipped to the LI‐6400. When used, the instrument was zeroed and the chemicals replaced (Meinzer et al., 2004). All measurements were conducted with the cuvette temperature set at 25°C and relative humidity at 60%.

### 2.2 Photosynthetic light‐response curves

Photosynthetic light‐response curves were calculated by gradually decreasing PPFD in 15 levels from 2,000 to 0 μmol m^−2^ s^−1^. In the meantime, the cuvette CO_2_ partial pressure was fixed at a constant level of 400 μmol/mol with a high‐pressure liquefied CO_2_ cartridge source. There was a 2‐min waiting period between each step, and measurements were taken when stability was achieved. Two light‐response curves were determined for each *P. massoniana* trees in the selected damaged stages for a total of 18 curves. *P*_max_, dark respiration rate, light compensating points (LCP), and light saturation points (LSP) were calculated from these light‐response curves using nonlinear regression techniques (Cavatte et al., 2012; Ögren & Evans, 1993). In addition, we calculated light‐use efficiency (LUE), instantaneous water‐use efficiency (WUE_i_), instantaneous carboxylation efficiency (CE_i_), and nitrogen‐use efficiency (NUE_i_), which are related to photosynthetic capacity and resource availability (Field et al., 1983; Hsu et al., 2015). The formulas are as follows:

$$\text{LUE}=P_{n}/\text{PPFD}$$

$${\text{WUE}}_{i}=P_{n}/E$$

$${\text{CE}}_{i}=P_{n}/C_{i}$$

$${\text{NE}}_{i}=P_{\max}/N_{\mathrm{mass}}$$

where *P*_n_ is the net photosynthetic capacity (μmol CO_2_ m^−2^ s^−1^), *P*_max_ is the greatest net photosynthetic value (μmol CO_2_ m^−2^ s^−1^), PPFD is the photosynthetic photon flux density(μmol m^−2^ s^−1^), *T* is the transpiration (μmol H_2_O m^−2^ s^−1^), *C*_i_ is the internal CO_2_ concentrations, and *N*_mass_ is foliar N content.

### 2.3 Carbon and nitrogen isotope ratio

After gas exchange was measured, foliar tissue (one sample for each branch) on which gas exchange was measured was collected, dried, and ground to a fine powder (Meinzer et al., 2004), and analyzed for δ^13^C, C%, and N% with an isotope ratio mass spectrometer (IsoPrime100, IsoPrime Corporation, Manchester, UK). The formula is as follow:

$$\delta_{\mathrm{sample}}\left(‰\right)=\left[\frac{R_{\mathrm{sample}}-R_{\mathrm{standard}}}{R_{\mathrm{standar}}}\right]\times 1000$$

where δ_sample_ is the value of δ^13^C of the measured foliar tissue samples, *R*_sample_ is the ^13^C/^12^C value of the measured sample, and *R*_standard_ is the ^13^C/^12^C value of the standard sample. The standard sample for ^13^C is Pee Dee Belemnite (PDB).

### 2.4 Statistical analyses

Photosynthetic data were collected and analyzed statistically using one‐way ANOVA. The Fisher's least significant difference (LSD) test with an alpha value of *p *<* *.05 level was used to compare the means of the photosynthetic characteristics, isotope abundance, and resource‐use efficiency of *P. massoniana* trees. A least squares linear regression model (again at *p *<* *.05) was used to assess the relationship between the foliar N content and δ^13^C, as well as *P*_max_, for each measured sample. All of the statistical analyses were performed using SPSS 22.0 for Windows (SPSS Inc., Chicago, IL, USA) and GraphPad Prism 6.0 (GraphPad Software, La Jolla, CA, USA).

## 3 Results

### 3.1 Effect on photosynthetic characteristics

**Figure 2:**
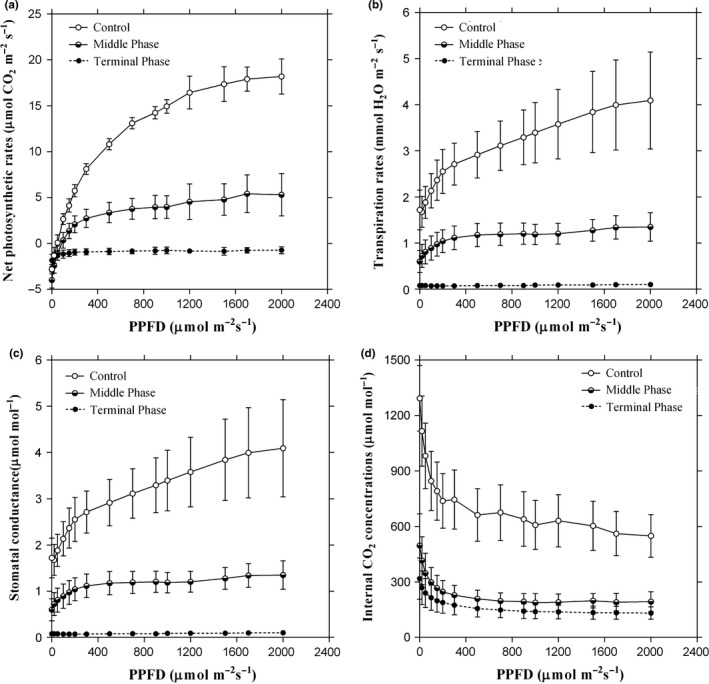
Changes in net photosynthetic rates (a), transpiration rate (b), stomatal conductance (c), and internal CO_2_ concentrations (d) among different phases of pine wilt disease‐infected Masson pine across the PPFD levels

From Figure 2a, we can clearly see that the *P*_n_ decreased in the infested plants at PPFD levels from 0 to 2,000 μmol CO_2_ m^−2^ s^−1^. In the infected trees, the *P*_n_ in the middle infection phase was higher than those in the terminal infection phase, for which the values were less than 0 across all PPFD levels. Similarly, the values of the *T*,* g*_s_, and *C*_i_ also decreased significantly (*p *<* *.05) in the infested *P. massoniana* plants as PPFD increased from 0 to 2,000 μmol m^−2^ s^−1^ when compared with the controls. (Figure 2b–d).

The values of *P*_max_ (*F *=* *190.07, *p *<* *.01) and LSP (*F *=* *26.906, *p *<* *.01) were significant decrease in the leaves of infested *P. massoniana* plants than those in controls (Table 1). However, the value of LCP was significantly higher in the infested treatment (*F *=* *8.141, *p *<* *.05, Table 1). The *P. massoniana* in the terminal infection phase did not have LSP and LCP because *P*_*n*_ is lower than 0. Significant differences in *R*_d_ exist among different treatments (*F *=* *146.459, *p *<* *.01, Table 1), but the visible trend is that measured photosynthetic values are highest during the middle infection phase, followed by the control and then the terminal infection phase, which has the lowest overall values.

### 3.2 Effect on carbon and nitrogen isotopic composition

*Pinus massoniana* trees show a significantly higher C:N in infested treatments (*F *=* *14.114, *p *<* *.01, Table 1). The difference was due to changes in foliar N mass concentration in infested Masson pines, for which the mean foliar N content was about 12.94% less in the middle infection phase and 27.06% less in the terminal infection phase when compared with those in control *P. massoniana* trees (Table 2). Conversely, there is little variation about the mean foliar C content (Table 2).

**Table 2:** Photosynthetic characteristics, nutrient content, and isotope abundance for foliage on intact branches of Masson pine trees in different phases of infection with pine wilt disease

Photosynthetic variables	Control	Middle phase	Terminal phase
*P*_max_ (μmol m^−2^ s^−1^)	18.71 ± 1.99 a	7.73 ± 2.21 b	−0.66 ± 0.32 c
*R*_d_ (μmol m^−2^ s^−1^)	2.81 ± 0.52 a	3.99 ± 0.87 b	1.85 ± 0.67 c
LCP (μmol m^−2^ s^−1^)	59.33 ± 7.77 a	82 ± 17.84 b	
LSP (μmol m^−2^ s^−1^)	1202.67 ± 299.13 a	431.33 ± 207.84 b	
LUE_max_ (μmol/μmol)	0.03 ± 0.00 a	0.01 ± 0.00 b	−0.00 ± 0.00 c
WUE_max_ (μmo (H20) μmol^−1^(CO_2_))	6.38 ± 4.23 a	4.17 ± 1.81 a	−7.19 ± 3.32 b
CE_max_ (mol m^−2^ s^−1^)	0.048 ± 0.033 a	0.031 ± 0.014 a	−0.008 ± 0.006 b
NUE (μmol m^−2^ s^−1^/mg g^−1^)	11.11 ± 1.72 a	5.31 ± 1.74 b	−0.52 ± 0.22 c
C_mass_ (%)	51.52 ± 0.95 a	51.82 ± 0.68 a	51.41 ± 0.78 a
δ^13^C (‰)	−25.27 ± 0.63 a	−27.69 ± 0.56 b	−27.83 ± 0.50 b
*N*_mass_ (%)	1.70 ± 0.11 a	1.48 ± 0.17 b	1.24 ± 0.13 c
C:N	30.50 ± 2.16 a	35.32 ± 4.18 b	41.77 ± 4.32 c

Values are mean ± SD of six replicates. For each row, values with different letters are significantly different at *P *=* *.05.

The foliar δ^13^C values were decreased about 2.5‰ in the infested trees when compared with the control *P. massoniana* trees. The foliar δ^13^C did not differ between the middle and terminal infection phases. The *P*_max_ estimated from the light‐response curves at 400 μmol/mol CO_2_‐saturated irradiance were 18.71, 7.73, and −0.66 μmol m^−2^ s^−1^ for control, middle infection, and terminal infection phases, respectively. In addition, the values of foliar δ^13^C and *P*_max_ of the *P. massoniana* trees exhibited a significant positive relationship with foliar N content (Figure 2, Adj. *r* ^2^_(δ_^13^_C‐N)_ = 0.964, *p *<* *.001; Adj. *r* ^2^_(*P*n‐N)_ = 0.617, *p *<* *.001).

### 3.3 Effect on resource‐use efficiency

Compared with controls, the LUE decreased significantly in the infested *P. massoniana* trees (*p *<* *.05), and the value for the terminal infection phase is less than 0 (Figure 4a). The value of WUE for the infested *P. massoniana* trees decreased as PPFD increased from 0 to 2,000 μmol m^−2^ s^−1^ when compared with the controls, but there was no significant difference between the control and middle infection phases (Figure 4b). Similarly, there was no significant decrease in CE for the middle infection phase trees. However, the CE for the terminal infection phase, which was steady and less than 0 at every PPFD, was significantly decreased (*p *<* *.05) in comparison with the controls (Figure 4c). The foliar N content was significantly lower (*p *<* *.05) in photosynthetic leaves of infested *P. massoniana* trees than those in control trees (Table 1). Meanwhile, the NUE markedly decreased in the infested *P. massoniana* trees (Figure 4d).

## 4 Discussion

In PWD researches, few studies have focused on the relationship between PWD and photosynthetic response and resource‐use efficiency. The present work provides new insights into the significant physiological changes that *B. xylophilus* infestation causes as measured by leaf gas exchanges, foliar carbon isotope ratios, and resource‐use efficiency in *P. massoniana* trees.

In this study, the values of *P*_n_, *T*,* g*_s_, and *C*_i_ across PPFD levels in *P. massoniana* leaves were significantly lower in the trees infected with *B. xylophilus* than in controls, and the corresponding value in the terminal infection phase decreased even more markedly (Figure 2). In addition, the decreasing *P*_n_ and *T* in the infected *P. massoniana* treatments were associated with a reduction in *g*_s_. Therefore, we can presume that the decreases in *P*_n_ and *T* are caused by the closure of stomatal pores in infested *P. massoniana* leaves. When a healthy tree is infected, *B. xylophilus* can cause dysfunction in the xylem and tracheid, blocking the sap ascent and water transportation from the roots to the crown (Zhao et al., 2008). With the dehydration of the mesophyll cells, the stomatal pores will partially or completely close, inhibiting the exchange of water and CO_2_ between external and internal foliar cells (Bigot, Fontaine, Clement, & Vaillant‐Gaveau, 2007; Hsu et al., 2015), and leading to a decrease in *T* and *C*_i_. Meanwhile, with a water deficit in the infected foliar cells, photosynthetic enzymatic activity can be inactivated or experience a loss of Rubisco and other key Calvin cycle enzymes (Bigot et al., 2007), which in turn reduces the total net photosynthetic rates. These results were consistent with previous studies, which reported that *P*_n_, transpiration, and *g*_s_ are known to covary in a consistent manner in many species (Bigot et al., 2007; Farquhar & Caemmerer, 1982; Hsu et al., 2015; Meinzer et al., 2004). In addition, from Figure 2, we can see the closure of stomatal pores has a greater effect on CO_2_ (*C*_i_) than H_2_0 (transpiration), which is mainly due to the host foliar cells showing additional resistance associated with diffusion of CO_2_ than H_2_0 (Zhang, Feng, Cregg, & Schumann, 1997).

Because PWD infection has a significant influence on photosynthetic enzymatic activities, significant differences in *P*_max,_ LCP, and LSP across light levels exist among three PWD‐induced treatments (Table 1). At the same time, *R*_d_ in the middle infection phase is higher than those in the control and terminal infection phases, indicating that most of the photosynthetic products in the middle infection phase were consumed by plant respiration. This may be due to the *P. massoniana* plants needing more energy to support their defense system in these infection phases to prevent pine sawyer beetle feeding and PWD infection (Zhao et al., 2008). This assumption is supported by many previous findings, which reported changes in respiration and oxidative enzyme levels in response to environmental stress (Bigot et al., 2007; Lopes & Berger, 2001; Zangerl, Arntz, & Berenbaum, 1997). Zangerl et al. (1997) noted that respiration rates increased 19% in pierced‐damaged leaves of wild parsnip 2 h after treatment. Lopes and Berger (2001) also found that the dark respiration rate increased on diseased leaves, which were damaged by rust and anthracnose. Bigot et al. (2007) found glycolate oxidase and cleavage T protein levels were raised by herbicide stress on grapevine.

Light‐use efficiency and carboxylation efficiency are two important parameters for estimating plant productivity (Akmal & Janssens, 2004). In our research, LUE (*P*_n_/PPFD) and CE (*P*_n_/*C*_i_) were significantly lower in the PWD‐induced *P. massoniana* plants, which contributed to the significant decline in *P*_n_. These results can be explained by the fact that *P*_n_ is an instantaneous measure of Rubisco enzyme activity (Makoi, Chimphango, & Dakora, 2010), which is affected by the invasion of PWD. Therefore, we can conclude that the reduction of LUE and CE in the PWD‐induced trees is caused by the disruption of Rubisco performance. Also, this assumption is confirmed by previous studies (Nabity, Heng‐moss, & Higley, 2006; Walia et al., 2010).

**Figure 3:**
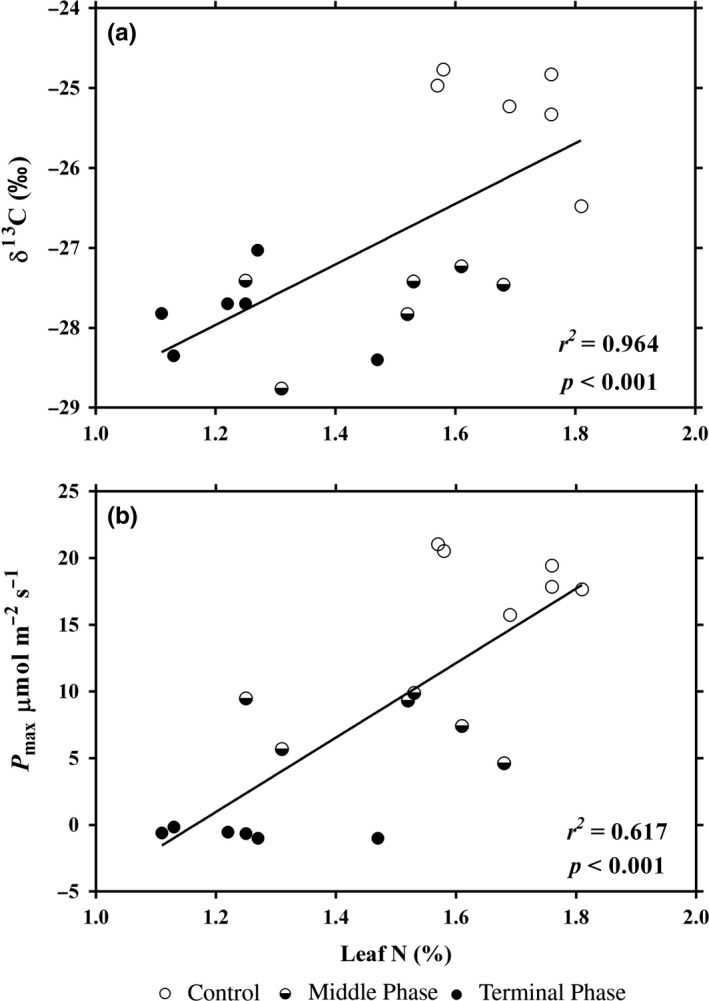
The Masson pine foliar δ^13^C values (a) and *P*_max_ estimated from the light‐response curves at 400 μmol/mol CO_2_‐saturated irradiance (b) in relation to foliar N content

**Figure 4:**
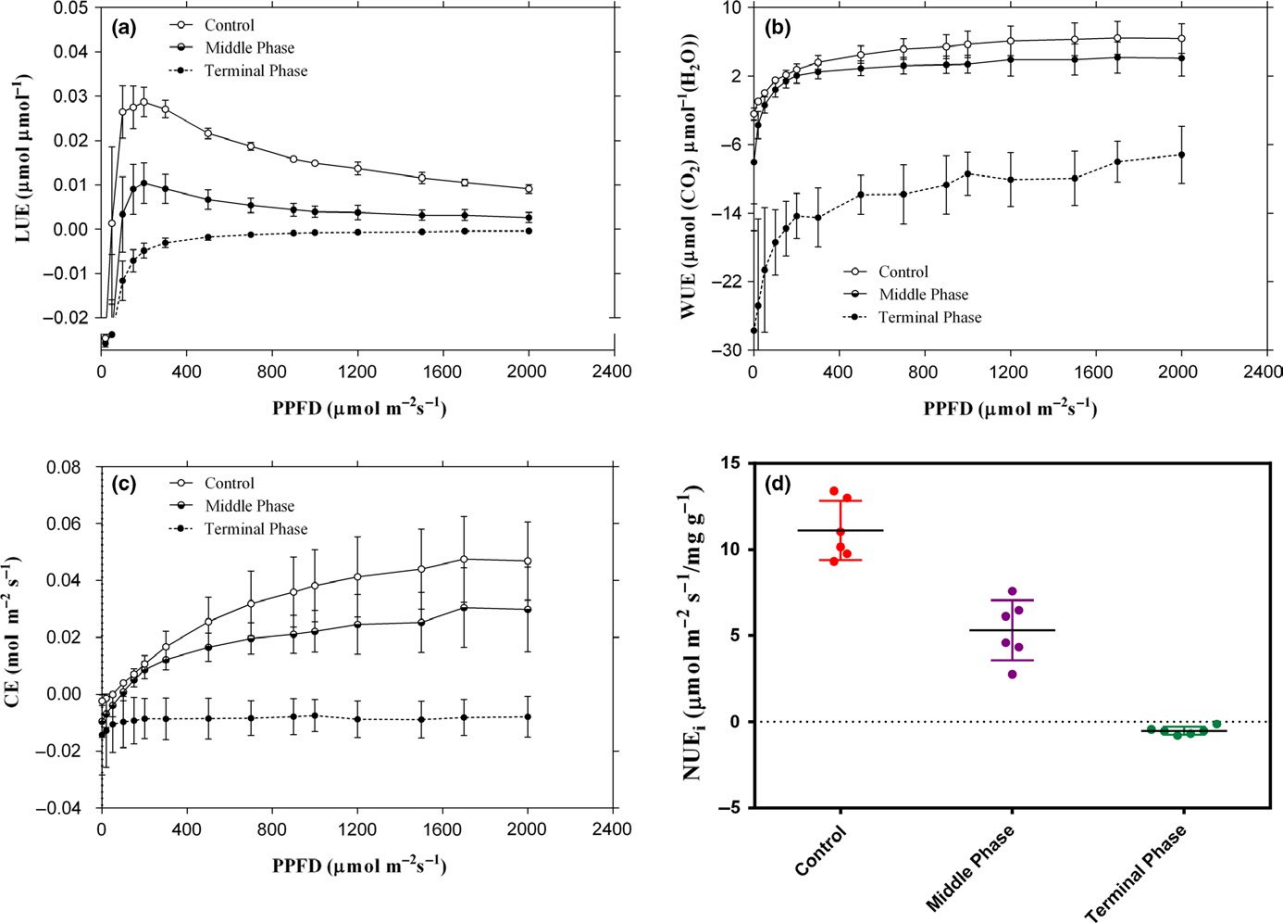
Changes in light‐use efficiency (a), water‐use efficiency (b), carboxylation efficiency (c), and nitrogen‐use efficiency (d) among different phases of pine wilt disease infection Masson pine across the PPFD levels

In our experiment, the foliar C:N ratio was significantly higher in PWD‐infected *P. massoniana* trees (Table 1), which is a result of a significant decrease in foliar N mass concentration in the infested Masson pines. The significant loss of foliar N content may be due to a lack of N uptake by the trees, caused by the closure of stomata. Similar results were found in dwarf mistletoe‐induced western hemlock, in which the leaf nitrogen content was 35% lower in infected trees (Meinzer et al., 2004). In 2007, Cabrera‐Bosquet also reported that plant nitrogen content was very susceptible to loss due to environmental stimulation (Cabrera‐Bosquet, Molero, Bort, Nogués, & Araus, 2007). In addition, the significant loss of foliar N content can influence the activity of the Rubisco enzyme that causes the reduction of photosynthetic capacity in infested *P. massoniana* trees (Clearwater & Meinzer, 2001; Walia et al., 2010; Warren, Dreyer, & Adams, 2003). In our data, *P*_max_ values were linearly correlated with the foliar N content of each measurement (Figure 3), which is consistent with existing literature. Similarly, NUE (*P*_max_/*N*_mass_) was also significantly lower in infected *P. massoniana* trees (Figure 4d).

δ^13^C is an important indicator that can represent the interactive relationship between plants and their surrounding biotic and abiotic environment factors (in this study, *P. massoniana* trees and *B. xylophilus*) (Dawson et al., 2002; Flower et al., 2013). In our research, the foliar δ^13^C level decreased as the PWD damage increased (Figure 3), which can be explained by a reduction of *g*_s_ and a decrease in the value of intercellular to ambient CO_2_ concentration (Cabrera‐Bosquet et al., 2007; Farquhar et al., 1989; Walia et al., 2010). This result was consistent with published studies, which reported a marked decrease in foliar δ^13^C values for conifers during a dwarf mistletoe infection (Meinzer et al., 2004; Sala, Carey, & Callaway, 2001).

Water is one of the most critical environmental factors that can affect the growth and performance of plants (Cavatte et al., 2012). WUE is defined as the amount of carbon gained per unit water loss (Sinclair, Tanner, & Bennett, 1984; Walia et al., 2010), which can be demonstrated directly by dividing *P*_n_ by *T* and indirectly by determining the δ^13^C value of foliar tissues (Zhang et al., 1997). In this study, the value of WUE for the infested *P. massoniana* trees decreased as PPFD increased from 0 to 2,000 μmol m^−2^ s^−1^ (Figure 4b); a significant decrease in foliar δ^13^C also occurred (Table 1), especially for the trees in the terminal infection stage. This result may be due to the fact that *P*_n_ did not change proportionally to PWD‐induced changes in transpiration, which have a more relative decrease of *P*_n_ and (or) a more relative increase of *T* in infested trees. These results were supported by previous studies, which reported the reduction of WUE in other infected hosts as indicated by a decline in foliar δ^13^C (Meinzer et al., 2004; Pataki, Oren, & Phillips, 1998).

In conclusion, our results demonstrate that *P. massoniana* trees undergo distinct photosynthetic changes and decrease in resource‐use efficiency with infection by PWD compared with uninfected trees. Our study suggests that the significant decreases in photosynthetic capacity and resource‐use efficiency of PWD‐infected *P. massoniana* trees are caused by the closure of stomatal pores and possibly also by the inactivation or loss of Rubisco and other key Calvin cycle enzymes. The results of this study will help forest managers to identify PWD infestations at the photosynthetic and physiological levels so as to better facilitate management actions.

## Conflict of Interest

None declared.

## References

[ece32642-bib-0001] Akmal, M. , & Janssens, M. J. J. (2004). Productivity and light use efficiency of perennial ryegrass with contrasting water and nitrogen supplies. Field Crops Research, 88, 143–155.

[ece32642-bib-0002] Bigot, A. , Fontaine, F. , Clement, C. , & Vaillant‐Gaveau, N. (2007). Effect of the herbicide flumioxazin on photosynthetic performance of grapevine (*Vitis vinifera* L.). Chemospehre, 67, 1243–1251. 10.1016/j.chemosphere.2006.10.079 17184818

[ece32642-bib-0003] Cabrera‐Bosquet, L. , Molero, G. , Bort, J. , Nogués, S. , & Araus, J. L. (2007). The combined effect of constant water deficit and nitrogen supply on WUE, NUE and Δ^13^C in durum wheat potted plants. Annals of Applied Biology, 151, 277–289.

[ece32642-bib-0004] Cavatte, P. C. , Oliveira, A. G. , Morais, L. E. , Martins, S. C. V. , Sanglard, M. P. V. , & DaMatta, F. M. (2012). Could shading reduce the negative impacts of drought on coffee? A morphophysiological analysis. Physiologia Plantarum, 144, 111–122. 21939445 10.1111/j.1399-3054.2011.01525.x

[ece32642-bib-0005] Clearwater, M. J. , & Meinzer, F. C. (2001). Relationships between hydraulic architecture and leaf photosynthetic capacity in nitrogen fertilized *Eucalyptus grandis* trees. Tree Physiology, 21, 683–690. 11446997 10.1093/treephys/21.10.683

[ece32642-bib-0006] Dawson, T. E. , Mambelli, S. , Plamboeck, A. H. , Templer, P. H. , & Tu, K. P. (2002). Stable isotopes in plant ecology. Annual Review of Ecology and Systematics, 33, 507–559.

[ece32642-bib-0007] Farquhar, G. D. , & Caemmerer, S. V. (1982). Modelling of photosynthetic response to environmental conditions. Physiological Plant Ecology II. Springer, Berlin, 12, 549–587.

[ece32642-bib-0008] Farquhar, G. D. , Ehleringer, J. R. , & Hubick, K. T. (1989). Carbon isotope discrimination and photosynthesis. Annual Review of Plant Biology, 40, 503–537.

[ece32642-bib-0009] Field, C. , Merino, J. , & Mooney, H. A. (1983). Compromises between water‐use efficiency and nitrogen‐use efficiency in five species of California evergreens. Oecologia, 60, 384–389. 28310700 10.1007/BF00376856

[ece32642-bib-0010] Flower, C. E. , Knight, K. S. , Rebbeck, J. , & Gonzalez‐Meler, M. A. (2013). The relationship between the emerald ash borer (*Agrilus planipennis*) and ash (*Fraxinus* spp.) tree decline: Using visual canopy condition assessments and leaf isotope measurements to assess pest damage. Forest Ecology and Management, 303, 143–147.

[ece32642-bib-0011] Fukuda, K. (1997). Physiological process of the symptom development and resistance mechanism in pine wilt disease. Journal of Forest Research, 2, 171–181.

[ece32642-bib-0012] Gao, R. H. , Shi, J. , Huang, R. F. , Wang, Z. , & Luo, Y. Q. (2015). Effects of pine wilt disease invasion on soil properties and Masson pine forest communities in the Three Gorges reservoir region, China. Ecology and Evolution, 5, 1702–1716. 25937913 10.1002/ece3.1326 PMC4409418

[ece32642-bib-0013] Hsu, M. H. , Chen, C. C. , Lin, K. H. , Huang, M. Y. , Yang, C. M. , & Huang, W. D. (2015). Photosynthetic responses of *Jatropha curcas* to spider mite injury. Photosynthetica, 53, 349–355.

[ece32642-bib-0014] Hu, G. , Xu, X. H. , Wang, Y. L. , Lu, G. , Feeley, K. J. , & Yu, M. J. (2012). Regeneration of different plant functional types in a Masson pine forest following pine wilt disease. PLoS One, 7, e36432. doi:10.1371/journal.pone.0036432 22563499 10.1371/journal.pone.0036432 PMC3341345

[ece32642-bib-0015] Kikuchi, T. , Jones, J. T. , Aikawa, T. , Kosaka, H. , & Ogura, N. (2004). A family of glycosyl hydrolase family 45 cellulases from the pine wood nematode *Bursaphelenchus xylophilus* . FEBS Letters, 572, 201–205. 15304348 10.1016/j.febslet.2004.07.039

[ece32642-bib-0016] Kim, C. , Jang, K. S. , Kim, J. B. , Byun, J. K. , Lee, C. H. , & Jeon, K. S. (2010). Relationship between soil properties and incidence of pine wilt disease and stand level. Landscape and Ecological Engineering, 6, 119–124.

[ece32642-bib-0017] Lopes, D. B. , & Berger, R. D. (2001). The effects of rust and anthracnose on the photosynthetic competence of diseased bean leaves. Phytopathology, 91, 212–220. 18944396 10.1094/PHYTO.2001.91.2.212

[ece32642-bib-0018] Makoi, J. H. J. R. , Chimphango, S. B. M. , & Dakora, F. D. (2010). Photosynthesis, water‐use efficiency and δ^13^C of five cowpea genotypes grown in mixed culture and at different densities with sorghum. Photosynthetica, 48, 143–155.

[ece32642-bib-0019] Mamiya, Y. (1988). History of pine wilt disease in Japan. Journal of Nematology, 20, 219–226. 19290205 PMC2618808

[ece32642-bib-0020] Meinzer, F. C. , Woodruff, D. R. , & Shaw, D. C. (2004). Integrated responses of hydraulic architecture, water and carbon relations of western hemlock to dwarf mistletoe infection. Plant, Cell and Environment, 27, 937–946.

[ece32642-bib-0021] Melakeberhan, H. , Toivonen, P. M. , Vidaver, W. E. , Webster, J. M. , & Dube, S. L. (1991). Effect of *Bursaphelenchus xylophilus* on the water potential and water‐splitting complex of photosystem II of *Pinus sylvestris* seedlings. Physiological and Molecular Plant Pathology, 38, 83–91.

[ece32642-bib-0022] Nabity, P. D. , Heng‐moss, T. M. , & Higley, L. G. (2006). Effects of insect herbivory on physiological and biochemical (oxidative enzyme) responses of the halophyte *Atriplex subspicata* (Chenopodiaceae). Environmental Entomology, 35, 1677–1689.

[ece32642-bib-0023] Ögren, E. , & Evans, J. R. (1993). Photosynthetic light‐response curves: I. The influence of CO_2_ partial pressure and leaf inversion. Planta, 189, 182–190.

[ece32642-bib-0024] Pataki, D. E. , Oren, R. , & Phillips, N. (1998). Responses of sap flux and stomatal conductance of *Pinus taeda* L. trees to stepwise reductions in leaf area. Journal of Experimental Botany, 49, 871–878.

[ece32642-bib-0025] Querejeta, J. I. , Barea, J. M. , Allen, M. F. , & Antonio, F. C. (2003). Differential response of δ^13^C and water use efficiency to arbuscular mycorrhizal infection in two aridland woody plant species. Oecologia, 135, 510–515. 16228249 10.1007/s00442-003-1209-4

[ece32642-bib-0026] Sala, A. , Carey, E. V. , & Callaway, R. M. (2001). Dwarf mistletoe affects whole‐tree water relations of Douglas fir and western larch primarily through changes in leaf to sapwood ratios. Oecologia, 126, 42–52. 10.1007/s004420000503 28547436

[ece32642-bib-0027] Shi, J. , Chen, F. , Luo, Y. Q. , Wang, Z. , & Xie, B. Y. (2013). First isolation of pine wood nematode from *Pinus tabuliformis* forests in China. Forest Pathology, 43, 59–66.

[ece32642-bib-0028] Shi, J. , Luo, Y. Q. , Song, J. Y. , Wu, H. W. , Wang, L. , & Wang, G. Z. (2007). Traits of Masson pine affecting attack of pine wood nematode. Journal of Integrative Plant Biology, 49, 1763–1771.

[ece32642-bib-0029] Shi, J. , Luo, Y. Q. , Song, J. , Yan, X. , Jiang, P. , & Wang, Y. (2006). Impact of the invasion of pine wood nematode and the following different removal disturbance intensity on the plant diversity of Masson pine community. Chinese Journal of Applied Ecology, 17, 1157–1163. 17044484

[ece32642-bib-0030] Sinclair, T. R. , Tanner, C. B. , & Bennett, J. M. (1984). Water‐use efficiency in crop production. BioScience, 34, 36–40.

[ece32642-bib-0031] Walia, A. , Guy, R. D. , & White, B. (2010). Carbon isotope discrimination in western hemlock and its relationship to mineral nutrition and growth. Tree Physiology, 30, 728–740. 20395303 10.1093/treephys/tpq020

[ece32642-bib-0032] Wan, F. H. , Zheng, X. B. , & Guo, J. Y. (2005). Biology and management of invasive alien species in agriculture and forestry. Beijing: Science publishing.

[ece32642-bib-0033] Warren, C. R. , Dreyer, E. , & Adams, M. A. (2003). Photosynthesis‐Rubisco relationships in foliage of *Pinus sylvestris* in response to nitrogen supply and the proposed role of Rubisco and amino acids as nitrogen stores. Trees, 17, 359–366.

[ece32642-bib-0034] Woo, K. S. , Yoon, J. H. , Woo, S. Y. , Lee, S. H. , Han, S. U. , Saeng, H. H. , & Kim, C. S. (2010). Comparison in disease development and gas exchange rate of *Pinus densiflora* seedlings artificially inoculated with *Bursaphelenchus xylophilus* and *B. mucronatus* . Forest Science and Technology, 6, 110–117.

[ece32642-bib-0035] Yoshimura, A. , Kawasaki, K. , Takasu, F. , Togashi, K. , Futai, K. , & Shigesada, N. (1999). Modeling the spread of pine wilt disease caused by nematodes with pine sawyers as vector. Ecology, 80, 1691–1702.

[ece32642-bib-0036] Yu, M. J. , Xu, X. H. , & Ding, P. (2011). Economic loss versus ecological gain: The outbreaks of invaded pinewood nematode in China. Biological Invasions, 13, 1283–1290.

[ece32642-bib-0037] Zangerl, A. R. , Arntz, A. M. , & Berenbaum, M. R . (1997). Physiological price of an induced chemical defense: Photosynthesis, respiration, biosynthesis, and growth. Oecologia, 109, 433–441. 28307541 10.1007/s004420050103

[ece32642-bib-0038] Zhang, J. W. , Feng, Z. , Cregg, B. M. , & Schumann, C. M. (1997). Carbon isotopic composition, gas exchange, and growth of three populations of ponderosa pine differing in drought tolerance. Tree Physiology, 17, 461–466. 14759838 10.1093/treephys/17.7.461

[ece32642-bib-0039] Zhao, B. G. , Futai, K. , Sutherland, J. R. , & Takeuchi, Y. (2008). Pine wilt disease. Tokyo: Springer.

